# Knowledge, Attitudes, and Practices towards Food Poisoning among Parents in Aseer Region, Southwestern Saudi Arabia

**DOI:** 10.3390/healthcare9121650

**Published:** 2021-11-28

**Authors:** Ayed A. Shati, Saleh M. Al Qahtani, Shehata F. Shehata, Youssef A. Alqahtani, Mohammed S. Aldarami, Sultan A. Alqahtani, Yahya M. Alqahtani, Aesha F. Siddiqui, Shamsun N. Khalil

**Affiliations:** 1 Department of Child Health, College of Medicine, King Khalid University, Abha 62529, Saudi Arabia; smuadi@kku.edu.sa (S.M.A.Q.); Youssef9811@hotmail.com (Y.A.A.); 2 Department of Family and Community Medicine, College of Medicine, King Khalid University, Abha 62529, Saudi Arabia; shfaraj@kku.edu.sa (S.F.S.); draeshasiddiqui@gmail.com (A.F.S.); shamsun203@gmail.com (S.N.K.); 3 Medical Student, College of Medicine, King Khalid University, Abha 62529, Saudi Arabia; msaldarami@gmail.com (M.S.A.); Sultan.1120@gmail.com (S.A.A.); y.albishri1@gmail.com (Y.M.A.)

**Keywords:** food poisoning, foodborne diseases, awareness, attitude, practice, Aseer, Saudi Arabia

## Abstract

**Background:** Food poisoning is caused by eating contaminated food. Improper food safety knowledge, poor food handling, and inadequate personal hygiene may allow microbes to grow in sufficient numbers to cause a food-borne illness. The aim of this paper was to assess the knowledge, attitudes, and practices regarding food poisoning and its determinants of parents in the Aseer region of Saudi Arabia. **Methodology:** This is a descriptive cross-sectional study conducted on 3011 parents in the Aseer region in the southwest region of Saudi Arabia. An online questionnaire was used to collect the data. The level of knowledge of the participants was scored as a percentage and further classified as “good” or “poor”. Attitudes were classified as “positive”, “neutral”, or “negative” based on a calculated composite mean score. The participants were asked about standard food hygiene practices, and the practices were recorded based on how often they were performed, with the results recorded as “usually”, “sometimes”, or “never”. **Results:** The age range of the parents was between 18 and 65 years old, with a mean age of 28.9 ± 10.4 years. Among the participants, 96.2% were Saudi, and 81.8% were female. Almost 53% of the fathers and 41% of the mothers were university graduates. About 55% of the mothers were housewives. Almost equal proportions of the parents had “good” and “poor” levels of knowledge on food poisoning. Around 41% of the parents had positive attitudes towards safe food consumption. Older parents (defined as above 30 years of age), males, university graduates, and urban residents had significantly higher levels of knowledge regarding food poisoning. **Conclusions:** This study provided much needed information on the knowledge, attitudes, and practices related to food poisoning among parents in the Aseer region of Saudi Arabia. Although most respondents reported satisfactory practices, gaps were identified in knowledge and attitudes. This suggests a need for further investigation focused on the observed practices and strengthening health education activities for the community.

## 1. Background

Foodborne illness (FBI), generally referred to as food poisoning, is caused by the consumption of food contaminated with bacteria, viruses, parasites, or other toxins [1,2]. The World Health Organization (WHO) reports that 1 in 10 people in the world fall ill after consuming contaminated food, and a good number of food poisoning cases occurs at home. About two million deaths occur annually due to food poisoning, especially in developing countries [3]. The burden of foodborne illness is shared among people of all age groups, particularly children, who contribute to one-third of the mortality rate for food poisoning [4]. In the United States, foodborne illnesses cause 76 million illnesses, 325,000 hospitalizations, and 5000 deaths yearly [5]. The Eastern Mediterranean region has the third-highest estimated burden of food-borne diseases per population, with more than 100 million people estimated to become ill with a food-borne disease every year. [4] According to the Ministry of Health data, there were 358 incidents of food poisoning reported in 2018 in Saudi Arabia [6].

The occurrence of foodborne illness depends on various factors. Food production, handling, and storage practices greatly influence the risk of foodborne illness, coupled with the personal hygiene practices of food consumers [7]. About half of the cases of food-borne illness are related to improper food storage or reheating practices, and the other half are related to issues with cross-contamination [8]. The risk of FBI is also influenced by factors such as gender, age, education, income level, and cultural factors [9]. Improper knowledge, attitudes, and practices towards food handling can lead to foodborne disease outbreaks. Food handlers at home are the main source of food poisoning in children [10]. Parents of young children carry the responsibility of providing safe meals to them. Hence, proper knowledge, attitudes, and practices are essential in maintaining food safety in the home. No studies about knowledge, attitudes, and practices of parents regarding food poisoning have been conducted in Saudi Arabia. This has caused an information gap for this important public health issue. This study aims to assess parents’ knowledge, attitudes, and practices regarding food poisoning in the Aseer region in southwest Saudi Arabia. It is expected that the findings of this study will enable policymakers and other stakeholders to set appropriate, evidence-based priorities in the area of food safety and to develop appropriate health education programs.

## 2. Methodology

### 2.1. Study Design, Procedure, and Participants

A descriptive cross-sectional study was conducted targeting all parents in the Aseer region of the southern part of Saudi Arabia. People 18 years and older with children living in the Aseer region were invited to participate in the survey. A total of 3500 eligible parents received the study survey, and 3011 parents completed the study questionnaire, with a response rate of 86%. Data were collected using an electronic questionnaire. The questionnaire was constructed after an intensive literature review and consultation with an expert.

A panel of three experts from the medical college of King Khalid University reviewed the questionnaire to check its clarity and content validity. Tool reliability was assessed using a pilot study of 25 participants with a reliability coefficient (α-Cronbach’s) of 0.73. The tool covered the following data: parents’ age, gender, education level, income, and their children’s ages. Knowledge in food poisoning was assessed using 15 questions. For the knowledge items, each correct answer was given one point, and the total sum of the discrete scores of the different items was calculated. Parents with a score of less than 9 points (corresponding to 60% of the maximum score) were considered to have poor knowledge of food poisoning. Good knowledge was considered to correspond with a score of 10 points or more.

The parents’ attitudes were assessed using 15 questions, and the parents’ practices were assessed using 20 questions that covered feeding habits and hygiene. For parent attitudes, the composite mean score was calculated for all the items, and the participants with a mean score below 1.5 were considered to have a negative attitude. Those with a composite mean score between 1.6 to 2.4 points were considered to have a neutral attitude. A positive attitude was assigned to those with a composite mean score of 2.5 to 3 points [11]. Standard food hygiene practices were inquired about and recorded as being practiced “usually”, “sometimes”, or “never”, measured as a percentage. The online questionnaire was uploaded on social media platforms, and all eligible parents were invited to complete the questionnaire after the purpose and confidentiality of the data were explained.

This research was approved by the Research Ethics Committee of King Khalid University (HAPO-06-B-001) on 5 June 2020 with the approval number ECM#2020-0706.

### 2.2. Data Analysis

After the data were extracted, it was revised, coded, and fed to the statistical software IBM SPSS version 22 (IBM Inc. Armonk, NY, USA). All statistical analysis was done using two-tailed tests. A *p*-value of less than 0.05 was considered to be statistically significant. Cross tabulation was used to assess the distribution of awareness according to participants’ bio-demographic data. Relations were tested using the Pearson chi-square test. Multiple logistic regression was undertaken to identify the factors.

## 3. Results

The study included 3011 participants whose ages ranged from 18 to 65 years old, with a mean age of 28.9 ± 10.4 years. The majority of the participants were females (81.8%), and 96.2% of the participants were Saudi. A total of 52.7% of the parents were university graduates, and 21.3% had an educational level below secondary school. Looking at the educational levels of the mothers only, 41.5% were university graduates, and 19.1% had an educational level below secondary school. About 55% of the mothers were housewives. A total of 81% of the participants were urban residents, and 37% had a monthly income of less than SR 10,000 (USD 2667). Almost 65.4% of the respondents had families of six people or more, and 23.2% of the participants only had one child. The mother was responsible for cooking in the home among 87.1% of the respondents, and 93.3% of the respondents had outdoor meals (Table 1).

Table 2 shows the parents’ knowledge regarding food poisoning. Around 85% of the respondents agreed that eating raw, unwashed vegetables is an extremely risky behavior that may lead to food poisoning, and 80.9% of respondents agreed that food processors who display unsanitary food practices can be a source of microbial contamination leading to food poisoning. A total of 79.6% of respondents reported that eating unwashed fruits is risky for food poisoning. A total of 74.6% of respondents know that food poisoning is caused by pathogenic microbes, and 74.1% of respondents agreed that eating raw or undercooked meat is very risky for food poisoning. Only 39% of respondents reported that pasteurized milk can be drunk directly without any risk of food poisoning, and 48.7% of respondents agreed on the absence of risk of food poisoning from eating cooked leftovers kept in the refrigerator for 2 to 3 days. Good knowledge regarding food poisoning was detected among 1527 (50.7%) participants.

Table 3 illustrates parents’ attitudes towards safe food consumption and food poisoning. Exactly 67.6% of the respondents disagreed that there is no risk of disease from eating unwashed vegetables and herbs picked directly from the plant, and the same proportion of respondents disagreed that there is no risk of disease from eating raw meat from young animals. A total of 58.3% of respondents disagreed that the rainwater collected in the tank is safe to drink without any treatment, while 56.9% disagreed that raw eggs are healthier and more nutritious than cooked ones. On the other side, 81.1% of respondents agreed that washing hands with soap and water before preparing food is essential to prevent food poisoning. A total of 79.1% of the participants agreed that handwashing with soap and water before eating is essential to prevent food poisoning, and 77.5% of respondents agreed that the thorough washing of vegetables and fruits with tap water is essential to prevent food poisoning. Overall, a positive attitude towards safe food consumption and food poisoning was detected among 1229 (40.8%) participants, while 250 (8.3%) had a negative attitude.

Regarding parents’ practices for preventing food poisoning (Table 4), 86.8% of the participants reported that they wash their hands with water and soap after handling raw, unwashed vegetables, 84.5% of the participants reported that they wash their hands with water and soap after using the toilet, 81.2% of the participants reported that they wash their hands with soap and water before eating meals, 70.8% of the participants reported that they wash fresh vegetables and fruits in tap water before eating, and 61.6% of the participants reported that they wash their hands with soap and water before preparing food. Only 20.1% of the respondents eat food such as meat, rice, and soup by hand from a large bowl shared by many people, 13.8% of the respondents eat food from a seemingly unclean restaurant or cafeteria, 11.9% of the respondents pick up vegetables or herbs from plants and eat them without washing, and 11% of the respondents eat undercooked eggs.

Table 5 shows the distribution of parents’ knowledge regarding food poisoning by their bio-demographic data. Good knowledge was detected among 57.9% of the parents above the age of 30 years compared to 46.8% of those below the age of 30 years, with a statistical significance (*p* = 0.001). A total of 60% of the male parents had a good knowledge level compared to 48.7% of the female parents (*p* = 0.001). A total of 52.9% of the university graduate fathers had good knowledge compared to 38.5% of the illiterate fathers (*p* = 0.003). Good knowledge was detected among 55.3% of the university graduate mothers compared to 45.2% of the illiterate mothers (*p* = 0.001). Good knowledge was significantly higher amongst working mothers than housewives at 55.2% and 47.7%, respectively. A total of 51.8% of urban resident participants had a good knowledge level compared to 46% of the rural residents (*p* = 0.012). Exactly of 56% of the participants who had outdoor meals more than twice per week had good knowledge compared to 42.6% of those who did not (*p* = 0.007).

The regression model shown in Table 6 shows that age was associated with a higher knowledge level, which was 2% higher for every additional year (OR (odds ratio) = 1.02; 95% CI (confidence intervals): 1.02–1.03). Female parents had a 35% lower knowledge level than the male parents (OR = 0.65; 95% CI: 0.54–0.78). Higher education for the mother was associated with an increased knowledge level of about 9% (OR = 1.09; 95% CI: 1.01–1.17). Housewives had 11% less knowledge than working mothers (OR = 0.89; 95% CI: 0.82–0.97). Parents who had a housekeeper for cooking showed a 16% lower knowledge level than the parents who cook by themselves (OR = 0.84; 95% CI: 0.75–0.93). Parents who had more frequent outdoor meals showed a 13% higher knowledge level regarding food poisoning than parents who did not have frequent outdoor meals (OR = 1.13; 95% CI: 1.04–1.23).

## 4. Discussion

Proper food handling knowledge and practices can go a long way in avoiding foodborne illnesses. The results of the current study reveal important information on the knowledge, attitudes, and practices of parents regarding food poisoning. Most of the respondents were Saudi females and were the main person responsible for cooking at home. This reflects a cultural bias towards cooking and food handling by women in the Arab region [12,13].

Our study observed that about half of the participants had good knowledge regarding the causes, risk factors, and prevention of food poisoning. The other half had poor knowledge scores. An analysis of the responses to the core statements revealed that most of the participants agreed that eating raw, unwashed vegetables is extremely risky in regard to food poisoning, food handlers can be a source of the microbial contamination of food, and eating unwashed fruits is risky for food poisoning. Regarding milk, more than 60% of the participants were unaware that pasteurized milk can be drunk directly without any risk of food poisoning, and almost half of the participants agreed that the risk of food poisoning can be avoided from eating cooked leftovers kept in the refrigerator for 2–3 days.

The knowledge scores of our study participants are similar to the knowledge scores of mothers in this region and other Asian and African countries, where it was reported that more than half of the respondents possess good knowledge of food poisoning [14,15,16]. However, lower knowledge levels were reported from the bordering country Yemen [17], which has similar religious and cultural milieu. The difference in findings between this study and studies in Yemen may be due to lower literacy levels and poor socio-economic conditions among the Yemeni population.

The study revealed that only 40% of the parents had a positive attitude towards safe food consumption and food poisoning. Almost half of the parents believed the rainwater collected in a tank is safe to drink without any treatment and raw eggs are healthier and more nutritious than cooked eggs. A good proportion of the respondents had an inappropriate attitude towards raw milk consumption, whether it be goat milk, cow milk, or camel milk, and a third believed that raw milk is healthier than pasteurized or boiled milk. A similar attitude towards the consumption of milk directly from the udder was also observed. The microbial contamination of milk and dairy products constitutes an important health risk for the consumers. Despite the latest reports implicating the practice of consuming raw milk as the most frequent source of exposure among patients with brucellosis, such practices and attitudes are still prevalent in the Middle East and North Africa, as reflected in the findings of recent studies [18,19]. Traditional beliefs play an important role in influencing food-related attitudes and practices. An example of this influence is the belief that fresh local produce is healthier and more beneficial, as well as the misconception that the nutrients in milk are destroyed when the milk is boiled [18]. These beliefs reflect lingering cultural influences in the study population, which was largely nomadic and agrarian until a few decades back, and food practices such as raw milk consumption were common. Interestingly, new trends of health food in developed countries also promote these attitudes and practices [20,21]. Focusing on the parents’ practices regarding food poisoning, three areas of concern were investigated: washing practices, eating raw foods, and storing and consuming food. We found satisfactory practices related to washing hands before and after food handling and consumption. The majority of the participants reported that they wash their hands with soap and water after handling raw, unwashed vegetables, after using the toilet, and before eating food. A good number of the participants also reported that they wash fresh vegetables and fruits in tap water before eating them. However, the practice of washing hands with soap and water before preparing food was not as common. The consumption of any form of raw food originating from an animal (i.e., milk, eggs, and meat) was only reported by a few participants. Other unhygienic practices, for example, eating foods such as meat, rice, and soup by hand from a large bowl shared by many people and eating food from a seemingly unclean restaurant or cafeteria, were also negligible.

Appropriate food handling practices reported in our study are a result of the Islamic influence on hygiene practices [22]. A recently published systematic review from Malaysia reported good levels of knowledge, attitudes, and practices among different food handler groups in Malaysia, thus supporting this notion [23]. Findings on food handling practices from our study contrast with findings reported from other Asian countries such as India and Nepal [24,25]. These dissimilarities reflect the differences in socioeconomic status, culture, and rural habitat. The study found that older age, being male, having a university-level or higher education, the working status of the mother, urban living, and frequently eating outside had a significant association with good knowledge regarding food hygiene and food poisoning. Several studies have reported that a higher age and being male was more positively associated with food hygiene knowledge. However, most of these findings were taken from professional food handlers [26,27]. Regarding educational level, studies specifically for mothers have also shown a positive association with educational level and knowledge regarding food hygiene and food poisoning [28]. These findings are in accordance with the findings from a study in Palestine where it was reported that those with a high level of education had higher knowledge scores than those with a lower level of education. It was also found that those who lived in a city or a village reported higher scores than those living in camps [12]. It is evident that a higher education level leads to higher knowledge levels. Social media is popular in the Arab world and has a penetration rate reaching almost 80% in Saudi Arabia [29]. Research in Saudi Arabia has recommended using social media to impart better health education to the population, particularly among females [30]. The relationship between urban living and better knowledge may reflect on improved access to information via social media and other mass media.

Despite satisfactory findings regarding food handling practices, the knowledge and attitudes of the parents in our study are worrisome. Considering that 30% of deaths related to food poisoning occur in children below five years of age [4] and that parents are the main people responsible for providing safe food for children, the study findings have important implications. These findings serve as a baseline of information for program planners and policymakers in Saudi Arabia and support the strengthening of health education initiatives for parents and other food handlers.

### Strength and Limitation

The key strength of this study is that it is the first study in this region that focuses on the knowledge, attitudes, and practices of parents regarding food poisoning. The large sample size of this study is its strength, as the results can be generalized to the Saudi population. The study’s major limitation is that the data were collected via an online survey, and the practice was not observable and only as reported by respondents. Hence, social desirability bias cannot be ruled out. Due to the cross-sectional design of the study, the causal factors of knowledge, attitudes, and practices related to food poisoning cannot be identified.

## 5. Conclusions

This study provided much-needed information regarding the knowledge, attitudes, and practices related to food poisoning among parents in the Aseer region of Saudi Arabia. Although most of the respondents reported satisfactory practices, gaps were identified in knowledge and attitudes. This suggests a need for further research focused on observed food handling practices. A mixed-method research approach targeting the main food handlers in the family and community could identify the gaps and associated factors. To prevent food poisoning, it is necessary to improve the knowledge, attitudes, and practices of food handlers. Thus, it is imperative to devise strategies for designing awareness programs about food poisoning and safe food handling for all food handlers in the community.

## Figures and Tables

**Table 1:** Bio-demographic data of study participants.

Bio-Demographic Data		(*n*)	%
**Age in years**	<30 years	1946	64.6%
	>30 years	1065	35.4%
**Gender**	Male	547	18.2%
	Female	2464	81.8%
**Nationality**	Saudi	2898	96.2%
	Non-Saudi	113	3.8%
**Father’s education** **(*n* = 548)**	Illiterate	39	7.1%
	Primary	46	8.4%
	Intermediate	32	5.8%
	Secondary	100	18.2%
	University	289	52.7%
	Above university	52	9.5%
**Mother ‘s education** **(*n* = 2463)**	Illiterate	84	3.4%
	Primary	146	6.0%
	Intermediate	240	9.7%
	Secondary	521	21.1%
	University	1021	41.5%
	Above university	451	18.3%
**Mother’s occupation** **(*n* = 2463)**	Governmental	948	38.5%
	Private	162	6.6%
	Housewife	1353	54.9%
**Residence**	Urban	2439	81.0%
	Rural	572	19.0%
**Monthly income**	<5000 SR	370	12.3%
	5000–10,000 SR	745	24.7%
	10,000–15,000 SR	680	22.6%
	15,000–20,000 SR	546	18.1%
	>20,000 SR	670	22.3%
**Total family size**	1–2	99	3.3%
	3–5	943	31.3%
	6+	1969	65.4%
**Number of children**	1	698	23.2%
	2–3	735	24.4%
	4–5	830	27.6%
	6+	748	24.8%
**Person responsible for cooking at home**	Mother	2622	87.1%
	Father	32	1.1%
	Housekeeper	357	11.9%
**Frequency of outdoor meals**	None	202	6.7%
	1–3/month	1279	42.5%
	1–2/week	953	31.7%
	>2/week	577	19.2%

SR: Saudi Riyal.

**Table 2:** Parents’ knowledge regarding food poisoning.

Knowledge Items	Yes		No		Don’t Know	
	(*n*)	%	(*n*)	%	(*n*)	%
Pathogenic microbes cause food poisoning.	2245	74.6%	130	4.3%	636	21.1%
Some toxins that microbes produce that cause food poisoning are resistant to the heating temperature of food.	1700	56.5%	530	17.6%	781	25.9%
Drinking raw milk is very risky for food poisoning.	1600	53.1%	496	16.5%	915	30.4%
Eating raw eggs is very risky for food poisoning.	1919	63.7%	567	18.8%	525	17.4%
Eating raw or undercooked meat is very risky for food poisoning.	2231	74.1%	403	13.4%	377	12.5%
Eating raw, unwashed vegetables is extremely risky for food poisoning.	2550	84.7%	257	8.5%	204	6.8%
Eating unwashed fruits is risky for food poisoning.	2396	79.6%	352	11.7%	263	8.7%
Food processors who use unsanitary practices can be the source of microbial contamination of food that causes food poisoning	2436	80.9%	185	6.1%	390	13.0%
Well-cooked food is free from microbes that cause food poisoning.	1805	59.9%	742	24.6%	464	15.4%
Eating uncooked cooked food, which is kept at room temperature for 12–24 h, is at high risk of causing food poisoning.	1579	52.4%	805	26.7%	627	20.8%
Raw white cheese manufactured from raw milk has a high risk of food poisoning.	1207	40.1%	550	18.3%	1254	41.6%
Pasteurized milk can be drunk directly without any risk of food poisoning.	1173	39.0%	573	19.0%	1265	42.0%
Keeping food at refrigerator temperature will slow microbial growth and beating, thus preventing food spoilage and food poisoning.	2203	73.2%	346	11.5%	462	15.3%
Drinking surface water such as rivers, streams, and rainwater tanks without any treatment, such as boiling or adding chlorine, is at great risk of causing food poisoning.	2092	69.5%	447	14.8%	472	15.7%
There is no risk of food poisoning from eating cooked leftovers kept in the refrigerator for 2–3 days.	1466	48.7%	1006	33.4%	539	17.9%
Overall knowledge level	Poor 1484 (49.3%)			Good 1527 (50.7%)		

**Table 3:** Parents’ attitudes towards safe food consumption.

Attitude towards Safe Food Consumption	Agree		Disagree		Don’t Know	
	(*n*)	%	(*n*)	%	(*n*)	%
Raw milk is healthier than pasteurized or boiled milk.	922	30.6%	980	32.5%	1109	36.8%
There is no risk of disease from drinking raw goat or cow’s milk immediately after milking.	808	26.8%	1617	53.7%	586	19.5%
There is no risk of disease from drinking camel’s milk right after milking.	1019	33.8%	1318	43.8%	674	22.4%
Raw eggs are healthier and more nutritious than cooked eggs.	606	20.1%	1714	56.9%	691	22.9%
There is no risk of disease from eating raw eggs.	620	20.6%	1728	57.4%	663	22.0%
There is no risk of disease from eating the raw meat of young animals.	458	15.2%	2034	67.6%	519	17.2%
Wiping fruits and vegetables makes them safe to eat.	965	32.0%	1635	54.3%	411	13.6%
There is no risk of disease from eating cooked food kept at room temperature for one day if covered.	1112	36.9%	1401	46.5%	498	16.5%
There is no risk of disease from eating unwashed vegetables and herbs picked directly from the plant.	576	19.1%	2036	67.6%	399	13.3%
If a child’s stool is free of pathogenic microbes, then he is not sick.	566	18.8%	1414	47.0%	1031	34.2%
The rainwater collected in the tank is safe to drink without any treatment.	640	21.3%	1756	58.3%	615	20.4%
Food processors without clinical symptoms can contaminate food with disease-causing microbes that cause food poisoning.	1364	45.3%	871	28.9%	776	25.8%
Handwashing with soap and water before eating is essential to prevent food poisoning.	2383	79.1%	326	10.8%	302	10.0%
Thorough washing of vegetables and fruits in tap water is essential to prevent food poisoning.	2335	77.5%	349	11.6%	327	10.9%
Washing hands with soap and water before preparing food is essential to prevent food poisoning.	2443	81.1%	274	9.1%	294	9.8%
**Overall attitude**	**Negative** 250 (8.3%)		**Neutral** 1532 (50.9%)		**Positive** 1229 (40.8%)	

**Table 4:** Parents’ practices regarding food poisoning.

Practice Items	Usually		Sometimes		Never	
	(*n*)	%	(*n*)	%	(*n*)	%
Do you wash fresh vegetables and fruits in tap water before eating?	2131	70.8%	794	26.4%	86	2.9%
Do you wash your hands with soap and water before eating your meal?	2444	81.2%	469	15.6%	98	3.3%
Do you wash your hands with soap and water before preparing food?	1855	61.6%	916	30.4%	240	8.0%
Do you wash your hands with soap and water after handling raw, unwashed vegetables?	2615	86.8%	288	9.6%	108	3.6%
Do you wash your hands with soap and water after using the toilet?	2545	84.5%	338	11.2%	128	4.3%
Do you wash your hands after touching animals?	544	18.1%	708	23.5%	1759	58.4%
Do you eat fresh vegetables and fruits without washing?	1407	46.7%	767	25.5%	837	27.8%
Do you wipe fresh vegetables and fruits before you eat them?	526	17.5%	768	25.5%	1717	57.0%
When taking a field trip, do you pick up vegetables or herbs from plants and eat them without washing?	359	11.9%	530	17.6%	2122	70.5%
Do you eat raw eggs?	505	16.8%	911	30.3%	1595	53.0%
Do you eat undercooked eggs (soft yolk)?	331	11.0%	490	16.3%	2190	72.7%
Do you eat raw meat?	413	13.7%	719	23.9%	1879	62.4%
Do you eat undercooked meat (inside red)?	447	14.8%	595	19.8%	1969	65.4%
Do you drink raw cow or goat milk?	447	14.8%	593	19.7%	1971	65.5%
Do you drink raw camel milk?	432	14.3%	683	22.7%	1896	63.0%
Would you eat raw white cheese prepared from pasteurized raw UN milk?	588	19.5%	1210	40.2%	1213	40.3%
Do you eat food cooked at room temperature for more than 6 h without adequate heating?	433	14.4%	977	32.4%	1601	53.2%
Are you eating food from a seemingly unclean restaurant or cafeteria?	417	13.8%	628	20.9%	1966	65.3%
Do you drink rainwater that is collected in tanks or surface streams without any treatment?	1119	37.2%	1107	36.8%	785	26.1%
Do you eat food, such as meat, rice, and soup, by hand from a large bowl shared by many people?	605	20.1%	725	24.1%	1681	55.8%

**Table 5:** Distribution of parents’ knowledge regarding food poisoning by their bio-demographic data.

Bio-Demographic Data		Knowledge Level				*p*-Value
		Poor		Good		
		(*n*)	%	(*n*)	%	
**Age in years**	<30 years	1036	53.2%	910	46.8%	0.001 *
	>30 years	448	42.1%	617	57.9%	
**Gender**	Male	219	40.0%	328	60.0%	0.001 *
	Female	1265	51.3%	1199	48.7%	
**Father’s education** **(*n* = 548)**	Illiterate	24	61.5%	15	38.5%	0.003 *
	Primary	24	52.2%	22	47.8%	
	Intermediate	16	50.0%	16	50.0%	
	Secondary	44	44.0%	46	56.0%	
	University	136	47.1%	153	52.9%	
	Above university	26	50.0%	26	50.0%	
**Mother’s education** **(*n* = 2463)**	Illiterate	46	54.8%	38	45.2%	0.001 *
	Primary	80	54.8%	66	45.2%	
	Intermediate	118	49.2%	122	50.8%	
	Secondary	281	53.9%	240	46.1%	
	University	457	44.7%	564	55.3%	
	Above university	240	53.2%	211	46.8%	
**Mother’s occupation** **(*n* = 2463)**	Governmental	425	44.8%	523	55.2%	0.001 *
	Private	82	50.6%	80	49.4%	
	Housewife	708	52.3%	645	47.7%	
**Residence**	Urban	1175	48.2%	1264	51.8%	0.012 *
	Rural	309	54.0%	263	46.0%	
**Total family size**	1–2	60	60.6%	39	39.4%	0.060
	3–5	469	49.7%	474	50.3%	
	6+	955	48.5%	1014	51.5%	
**Number of children**	1	317	45.4%	381	54.6%	0.122
	2–3	370	50.3%	365	49.7%	
	4–5	425	51.2%	405	48.8%	
	6+	372	49.7%	376	50.3%	
**Person responsible for cooking at home**	Mother	1279	48.8%	1343	51.2%	0.128
	Father	13	40.6%	19	59.4%	
	Housekeeper	192	53.8%	165	46.2%	
**Frequency of outdoor meals**	None	116	57.4%	86	42.6%	0.007 *
	1–3/month	643	50.3%	636	49.7%	
	1–2/week	471	49.4%	482	50.6%	
	>2/week	254	44.0%	323	56.0%	

P: Pearson X2 test. * *p* < 0.05 (significant).

**Table 6:** Multiple logistic regression model for determinants of parents’ knowledge regarding food poisoning.

Factors	*p*-Value	OR_A_	95% CI	
			Lower	Upper
Age in years	0.001 *	1.02	1.02	1.03
Female vs. male parents	0.001 *	0.65	0.54	0.78
High mother education (university vs. others)	0.018 *	1.09	1.01	1.17
High father education (university vs. others)	0.304	0.97	0.90	1.03
Housewives vs. working mothers	0.009 *	0.89	0.82	0.97
Rural vs. urban residence	0.183	0.88	0.73	1.06
Family size	0.099	1.02	1.00	1.05
Number of children	0.086	0.98	0.96	1.00
Housekeeper cooking vs. parents	0.002 *	0.84	0.75	0.93
Frequency of outdoor meals	0.004 *	1.13	1.04	1.23

OR: odds ratio; CI: confidence intervals. * *p* < 0.05 (significant).

## Data Availability

The datasets used and analysed during the current study are available from the corresponding author on reasonable request.
